# Patients pathways to tuberculosis diagnosis and treatment in a fragmented health system: a qualitative study from a south Indian district

**DOI:** 10.1186/s12889-017-4627-7

**Published:** 2017-08-04

**Authors:** Vijayashree Yellappa, Pierre Lefèvre, Tullia Battaglioli, Narayanan Devadasan, Patrick Van der Stuyft

**Affiliations:** 1 Institute of Public Health, #250, 2nd C Main, 2nd ‘C’ Cross-, Girinagar I Phase, Bangalore, Karnataka 560 085 India; 2 Institute of Tropical Medicine, Nationalestraat, 155, 2000 Antwerp, Belgium; 3 Public Health Department, Faculty of Medicine, Ghent University, Ghent, Belgium

**Keywords:** Tuberculosis, Private practitioners, Referral practices, Therapeutic itinerary, Kickbacks, RNTCP, PPM, Private pharmacists, India

## Abstract

**Background:**

India’s Revised National Tuberculosis (TB) Control Programme (RNTCP) offers free TB diagnosis and treatment. But more than 50% of TB patients seek care from private practitioners (PPs), where TB is managed sub-optimally. In India, there is dearth of studies capturing experiences of TB patients when they navigate through health facilities to seek care. Also, there is less information available on how PPs make decisions to refer TB cases to RNTCP. We conducted this study to understand the factors influencing TB patient’s therapeutic itineraries to RNTCP and PP’s cross referral practices linked to RNTCP.

**Methods:**

We conducted in-depth interviews on a purposive sample of 33 TB patients and 38 PPs. Patients were categorised into three groups: those who reached RNTCP directly, those who were referred by PPs to RNTCP and patients who took DOT from PPs. We assessed patient’s experiences in each category and documented their journey from initial symptoms until they reached RNTCP, where they were diagnosed and started on treatment. PPs were categorised into three groups based on their TB case referrals to RNTCP: actively-referring, minimally-referring and non-referring.

**Results:**

Patients had limited awareness about TB. Patients switched from one provider to the other, since their symptoms were not relieved. A first group of patients, self-medicated by purchasing get rid drugs from private chemists over the counter, before seeking care. A second group sought care from government facilities and had simple itineraries. A third group who sought care from PPs, switched concurrently and/or iteratively from public and private providers in search for relief of symptoms causing important diagnostic delays. Eventually all patients reached RNTCP, diagnosed and started on treatment. PP’s cross-referral practices were influenced by patient’s paying capacity, familiarity with RNTCP, kickbacks from private labs and chemists, and even to get rid of TB patients. These trade-offs by PPs complicated patient’s itineraries to RNTCP.

**Conclusions:**

India aims to achieve universal health care for TB. Our study findings help RNTCP to develop initiatives to promote early detection of TB, by involving PPs and private chemists and establish effective referral systems from private sectors to RNTCP.

## Background

In India, Tuberculosis (TB) is a major public health problem, carrying one fourth of the global burden of TB [1]. Government of India launched Revised National TB Control Programme (RNTCP) in 1997 based on World Health Organisation (WHO) endorsed Directly Observed Treatment Strategy (DOT). The programme follows a ‘passive case finding’ approach, which assumes that people are able to recognise their symptoms and access health care in time. RNTCP is implemented mainly through government health facilities, which provide quality assured free diagnosis and treatment. TB patients can avail RNTCP services either accessing directly or being referred by Private Practitioners (PPs).

In spite of the availability of free public services, more than 50% of patients are estimated to seek care in the private health sector [2, 3]. But, studies have shown that the TB management practices in private sector is sub-optimal [4, 5]. Recognising the critical importance of PPs, RNTCP is involving them through Public Private Mix (PPM) strategy [6]. PPs can refer TB patients to RNTCP either for free diagnosis and/or treatment. However, PP’s involvement in RNTCP is meagre [7], despite the promising results of PPM strategy [8, 9].

Although RNTCP is successful in terms of increase in the proportion of patients treated, many TB cases are missed by RNTCP. In fact India tops the list of ten countries that account for 74%(2.4 million) of the estimated missed cases globally [1]. ‘Missed cases’ are those cases which are neither detected nor notified to RNTCP. This can be attributed to either patients delay in accessing healthcare or health system delays [10] or to PPs’ reluctance to notify cases to RNTCP [11]. Several studies in India have analysed health seeking behaviour of TB patients and the factors determining their decision-making [12, 13]. Much of the studies assessing diagnostic and treatment delays have aimed to quantify the delays [14, 15]. The determinants of these delays are much more complex and require a comprehensive understanding of patients’ journey from the onset of symptoms until they are successfully treated. But, there is scant published research in India that analyses TB patients experiences holistically, as they navigate through health facilities. Further, it is also critical that PP’s TB management practices, their cross-referral practices linked to RNTCP and patients’ personal beliefs be comprehended and corroborated.

With this background, we conducted this study among TB patients taking treatment in the RNTCP and PPs providing TB care in Tumkur district, a south Indian state. We aimed to understand patient’s therapeutic itineraries to RNTCP, factors influencing the itineraries and corroborated with PP’s cross referral practices linked to RNTCP.

Data presented in this paper were collected as part of a larger study, which investigated the operational challenges in establishing the collaboration between PPs and RNTCP in Karnataka state.

## Methods

### Study design

Qualitative research using in-depth interviews as data collection tool.

### Study setting

Tumkur district (population of 2.7 million, spread over 10,597 km^2^) has pluralistic health system composed of private and public health facilities. Public sector includes a district hospital, ten sub-district hospitals and 146 primary health centres. Private health sector is heterogeneous comprising of highly specialised PPs to informal providers, who practice modern medicine without any formal training. Retail private chemists often dispense drugs over-the counter. Sub-districts are responsible for TB programme implementation. Under each sub-district, there are designated microscopy centres, which provide free sputum smear microscopic services. District has 2555 DOT (Directly Observed Treatment) centers that provide free RNTCP TB drugs and ensure treatment completion. DOT provider can be a public health facility (all of them provide DOT), a PP, a health worker or any trained community volunteer who is acceptable and accessible to patients. Structure and functioning of RNTCP is elaborated elsewhere [16].

### Study participants and sampling

We conducted in-depth interviews with a purposive sample of TB patients aged 15 years and above (*n* = 33). Patients were stratified for rural (*n* = 18) and urban (*n* = 15) settings. Patients started on the RNTCP treatment were shortlisted from RNTCP laboratory registers, and categorised depending on how they reached RNTCP: (i) those who reached RNTCP directly by their own without any referral (ii) those referred by PPs to RNTCP for diagnosis and/or treatment and (iii) those who were diagnosed by RNTCP and referred to PPs for DOT. Patient details were collected from patient TB treatment cards and TB registers. Patients who had completed their treatment in the last 3 months or who were about to complete the treatment in the next 1 month at the time of interviews were selected to reduce recall bias. We aimed to interview eight respondents from each category to reflect the typical experiences of patients based on the principle of data saturation. Although we targeted eight respondents in each category, we interviewed more respondents in the category of ‘referred by PPs’ (*n* = 17). This is because, during the course of some interviews, we discovered that some patients who were categorised as ‘having reached RNTCP directly’ had initially consulted PPs and were referred by them to RNTCP.

PPs (*n* = 38) were selected purposively from the list (*n* = 198) maintained at the district TB centre. We collected number of presumptive TB case referrals made by PP’s to RNTCP for the year 2012 from RNTCP laboratory registers, and categorised PPs into three groups based on the referrals into: (i) actively-referring PP; who had referred 12 or more than 12 cases (*n* = 17) (ii) minimally-referring PP; who had referred at least one, but less than 12 cases (*n* = 8) and (iii) not-referring PP; who had not referred any case (*n* = 13). Though, we considered all the 19 actively-referring PPs, we could interview 17 of them, since two of them were out of town during the study period. Efforts were made to match non-referring and minimally-referring PPs with actively-referring PPs for age, qualification, patient load and location of practice.

### Data collection

Data were collected from May 2013 to August-2013. Six interview guides, one for each category of patients and PPs were pilot-tested in February 2013 and fine-tuned. Interviews were preceded by warm up visits to interviewees by the field co-ordinator and the principal investigator (PI) of the study (VY). The field coordinator shared the information brochure with patients in local language, Kannada. Similarly, the PI visited PPs and shared the information brochure in English. During these visits, an appointment was sought for interviews. All interviews were conducted by the PI and they were audio recorded.

Interviews with patients were conducted in Kannada that lasted 45 to 90 min. Of the 33 interviews, 23 participants chose to be interviewed at their residence and the remaining at DOT centres and near their working place. Family members were present in 21 of these interviews and helped patients to display laboratory reports and medical prescriptions wherever available. Of the 47 shortlisted patients, 10 refused to be interviewed, and four had moved out.

PP’s interviews were conducted in their health facilities, on an average we had to make three attempts to get their appointments. Interviews with PPs were mix of English and Kannada, which lasted 45 to 80 min. Interviews were transcribed verbatim by professional transcribers from the digital version. Each transcription was then crosschecked by the PI for accuracy. In addition, the note taker documented the main points raised, setting descriptions and any relevant informal conversations happened before and after the interviews.

### Data analysis

To maintain the confidentiality, personal details of participants were removed and audio files were anonymized. Data were analysed using a combination of deductive and inductive approaches. The deductive approach was based on the research questions, which primarily aimed to examine the factors influencing TB patient’s therapeutic itineraries to RNTCP and understand PP’s cross-referral practices to RNTCP. VY and PL devised a coding scheme jointly. Each transcript along with the field summary notes was coded by using NVIVO software version 9 (QSR International Pvt. LTD, Melbourne, Australia). These initial codes were tested on a handful of interviews, which were then refined and organised at a broader conceptual level into themes by grouping them together [17, 18]. We considered the flexibility of including new themes emerging from the data (inductive approach). This approach helped us to identify relationships and corroborate PP’s and patient’s views. We then conducted thematic analysis, and explored the relationships between and across the themes across different categories of participants [19]. Memos were written on patient’s navigation to RNTCP and PP’s cross referrals practices and the data were corroborated. To increase the internal validity of the analysis, the coding scheme was regularly discussed with a sociologist (PL) not involved in data collection and other members of the research team.

The analysis indicated the challenges that the patients face as they navigate through the fragmented health care settings; patient’s making sense of the symptoms and the disease, patient’s making decision to seek care, patient therapeutic itineraries until they were diagnosed with TB, PP’s TB management practices and how it is perceived by patients, how PP’s make a decision to refer cases to RNTCP and how it influences patient’s itineraries.

## Results

After summarising the socio-demographic characteristics of participants, we present the findings under the following major themes: first we present patients reported initial symptoms and their TB awareness. Then we illustrate patient’s itineraries from the onset of symptoms to TB diagnosis at RNTCP’s microscopy centres. Later we proceed to elaborate the factors that influence patient’s itineraries including PP’s TB management and their cross referral practices linked to RNTCP. In the following result section we elaborate these themes.

### Participant’s characteristics

Average age of patients was 42 years (range 18 to 70 years). Gender was equally represented. Reported income per month ranged from USD 46 to 229. All patients had pulmonary TB, expect three who had extra pulmonary TB and none of them took treatment for multidrug resistant TB. Average duration of PPs’ experience was 26 years (range 7 to 39 years). All PPs except three were male; 23 were from rural area and 15 from urban; 32 practiced allopathy (17 general practitioners and 15 specialists) and six were informal providers.

### Patients reported initial symptoms and their TB awareness

Excluding patients who had extra pulmonary TB, most frequently reported symptoms were cough associated with fever, tiredness and weight loss. Two patients spoke about coughing blood. Patient’s, awareness about TB seems to be limited to the fact that the disease is transmitted by cough and to be influenced by previous exposure of their relatives to TB. A number of patients stated quotations very similar to this one: “*No. I was not aware of anything about TB. I never knew anything about it. Nobody from my family or neighbours had this”* [Man, Rural, PP Referred]. Patients cited the following reasons as sources of TB transmission: getting exposed to TB patients and dust, living in unhygienic environment, alcohol consumption, smoking and eating junk food on roadside. Men mostly advanced the latter reasons. There were some other factors seldom cited by patients from rural settings such as starving, typhoid fever, walking on sputum or urine of a TB patient and even worms. People who had co-morbid conditions such as HIV or asthma, perceived TB as a lesser problem, because they thought TB could be cured, but not the former conditions.

### Patient’s therapeutic itineraries

**Fig. 1:**
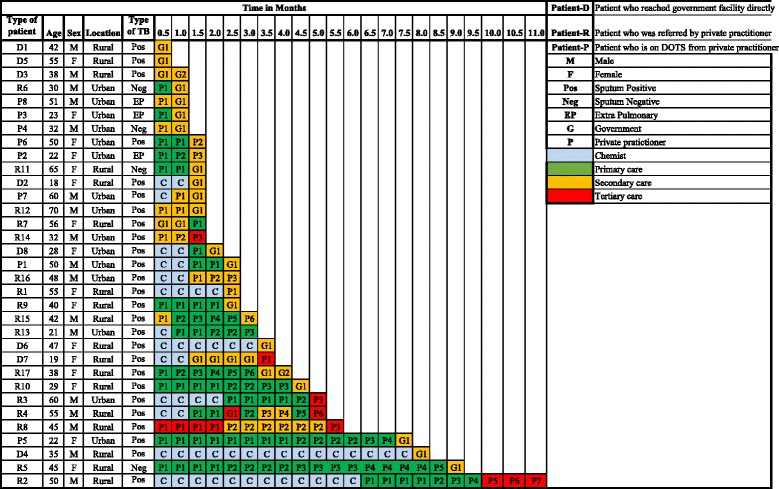
Therapeutic itineraries of tuberculosis patients

From the onset of symptoms, two main courses of action emerge from the data. In Fig. 1; Therapeutic itineraries of TB patients, we illustrate the navigation of 33 patients through various health care facilities from the onset of symptoms to TB diagnosis at RNTCP’s designated microscopy centres.

The first group of patients (*n* = 13) self-medicated by purchasing symptomatic drugs over the counter from nearby retail private chemists for a period varying from 2 weeks to seven and a half months. Two patients from this category, patient-D4 and patient-R2 encountered the longest delays before getting diagnosed with TB, up to 8 months and 11 months respectively (Fig. 1).

Of the eight patients from the ‘directly reached category’, three patients (D1, D3 and D5) sought care from the government facilities directly and they were diagnosed there with no delay. Remaining five patients sought care from private chemists initially, by purchasing symptomatic drugs over-the counter. Since the symptoms did not subside, they accessed RNTCP by themselves based on the following factors: (i) suggestions by the government field health workers, (ii) familiarity with government hospitals, and (iii) having had health personnel among their relatives. In general, this category of patients who reached RNTCP directly had simple itineraries, with least number of providers visited compared to the patients who first sought care from PPs (Fig. 1).

A third group, who sought care from PPs (*n* = 17), switched concurrently and/or iteratively from public and private providers in search for relief of symptoms causing important diagnostic delays (Fig. 1). Eventually all patients reached RNTCP and were started on treatment. Of the 17 PP referred cases, one patient had visited the government facility initially. Since patient’s symptoms were not relieved there, he sought care from a PP. He expressed his dissatisfaction about the poor services offered at government hospital. This concern was also raised by three PP referred patients (R4, R7, R17):

“*Government doctor did not show any interest, neither he responded to my questions. They never spoke to me at all. We went there 1–3 days and became fed-up. Even the 4* ^*th*^ *day they did not say anything. They asked me to go here and there. It was really a horrible experience to run around there. So, finally we decided and went to private*” [Man, Rural, PP referred].

More than half of the respondents approached primary level health facilities in the initial course of illness (either private or public). However, except one patient (R7), final diagnosis of TB was made at either secondary or tertiary level facilities after much itinerancy (Fig. 1).

### Factors influencing patient therapeutic itineraries

If we consider all patients, three factors played an important role influencing patient itineraries; (i) influence of significant others, (ii) PP’s TB management practices, (iii) PP’s cross referral practices linked to RNTCP (iv) provision of kickbacks and (v) patient’s search for relief.

#### Influence of significant others and preference for particular providers

More than half of the patients (*n* = 16), while discussing their therapeutic itineraries spoke about the role of the family, relatives, friends and even neighbours at some point of time. These interventions took various forms, ranging from suggesting the patient to go to a particular hospital or to change the provider, or even ‘force’ the patient to seek care. A quote:

“*She* [Daughter] *said that the symptoms sound like TB. So, we should go and get a check-up”* [Woman, Rural, Directly Reached].

We observed that the trust in family doctors played an important role in at least one third of patients in choosing a particular doctor. Patients esteemed these providers; “*He is our family doctor. Whatever happens in our family we will get treatment from him*” [Woman, Urban, Patient taking DOT from PP].

#### Provider’s TB management practices

TB management practices of PPs played an important role in influencing patient’s itineraries to RNTCP.

##### Low index of suspicion of TB among PPs

Indirect evidence of low index of suspicion of TB among PPs can be deduced from patients’ narratives. Some patients were misdiagnosed and treated for typhoid and dust allergy for a period ranging from 1 week to 3 months with no relief of symptoms. Patients were unhappy that PPs were unable to detect TB in the first instance and that they were made to undergo unnecessary tests. An excerpt:

“*He* [PP] *had told me to take injections daily and I was taking it as advised. But he did not tell me anything. He kept on saying it is typhoid. We told him that sputum is coming while coughing. But he said, it will happen like this even for typhoid also*” [Woman, Rural, PP referred].

This was conceded by some PPs, who expressed how increasingly it is becoming difficult to diagnose TB because of varied clinical presentations associated with HIV and diabetes. PPs highlighted the difficulties they face to diagnose extra-pulmonary TB and sputum negative pulmonary TB cases.

##### PP’s TB management practices

PPs used a battery of investigations to diagnose TB, such as chest X-ray, blood tests, sputum examination, and Mantoux test by order of preference. Chest X-ray was most preferred diagnostic tool, since PP’s perceived it to be rapid and patient friendly. An excerpt:

*“X-ray is fast. We get the result in 10–15 minutes and immediately we can take some decisions. You know… we do not want to miss any patients* (smiles)” [Actively-Referring PP, Rural, Specialist].

Majority of PPs tend to reject X-rays taken at the government hospitals, as they perceived them to be of poor quality. Thus, patients were asked to repeat the chest X-rays in private labs, which meant double expenditure and investment of time for patients. A quote:

“*I don’t trust government hospital for X-ray. Quality of X-ray is not good there. X-ray technician there* [Government hospital] *is not efficient. Only if patients refuse to go to private X-ray facility, we will send them to government for X-ray*” [Actively-Referring PP, Rural, General Practitioner].

Contrastingly, all the actively-referring PPs preferred government hospitals for sputum microscopy. They placed great trust on the technical competency of government laboratory technicians and believed that the technicians are regularly trained as compared to private ones. They listed several advantages of ordering sputum examination compared to other TB tests: being specific for TB, easier to communicate the TB diagnosis to patients when sputum results are positive and helps in monitoring the disease condition. This group of PPs were familiar about RNTCP and referred most of their cases to RNTCP for sputum examination. In some instances when patients refused to go there, because of perceived low quality of care, PPs counselled them. A quote:

*“RNTCP lab technicians are well trained. They always give good results. When some patients insist that they will get the tests done from private labs, I tell them. “There* [private lab] *they* [lab technicians] *will not do it well”. We insist them* [patients] *to go to government only. I never send my patients to private labs for sputum AFB* [Acid Fast Bacilli]*”* [Actively-Referring PP, Rural, General Practitioner].

As far as the TB treatment practices were concerned, actively-referring PPs preferred to refer cases to the RNTCP. In instances where patients refused RNTCP treatment, they were started on private treatment with daily regimen, four anti-TB drugs for initial 2 months, followed by two anti-TB drugs for 4 months. Minimally-involved and non-involved PPs either treated TB patients sub-optimally for 3 months or over-treated for a year and a half, with inappropriate regimen. Antibiotic Oflaxacin was mostly combined with anti-TB treatment regimens. All PPs except one (Non-referring PP-4) acknowledged that, patients tend to default after 2 months of private treatment once the symptoms subside. They also stated that they cannot follow up patients to ensure treatment completion. Informal providers also treated TB patients with inappropriate regimen for 3–4 months. Their TB prescription knowledge was mainly based on medical representatives visits and the drugs they propose. When patient’s symptoms did not subside, they tend to refer them to specialists located in district head quarter.

##### PP’s cross referral practices linked to RNTCP

The decision to refer patient to RNTCP was solely dependent on PPs’ discretion. Most of the actively-referring PPs were familiar with the RNTCP and preferred to refer their cases to RNTCP. Some of them even believed that, it was their duty to co-operate with government, since most of them had received subsidised medical education from the government. Others expressed that referring patients to RNTCP was a social service. A quote:

*“My patients are poor. They cannot afford private treatment. So I refer them to TB hospital. I feel it is a social service that I am doing”* [Actively referring PP, Urban, Specialist].

However, minimally-referring and non-referring PPs’ decision to refer patients to the RNTCP was solely dependent on patient’s paying capacity. They referred only such patients to the RNTCP, whom they perceived of incapable of paying.

*“I will be knowing their* [patient’s] *financial condition. I can make out whether they are affordable or not. If they are unaffordable, I will ask them to get the sputum test done in government”* [Non-Referring PP, Urban, Specialist].

Contrastingly, they retained such patients who had the capacity to pay for private treatment. We observed that some minimally-referring PPs referred patients to the RNTCP to get rid of them. They perceived TB patients as nuisance, since TB patients bothered them frequently with small ailments and repeated counselling consumed lot of their time. In some instances, when PPs sensed that they could not ensure patient’s compliance to treatment, they referred such patients to RNTCP. A quote:

*“I do not have time to sit and explain to the patients, about what is TB and all. This communication is very time consuming. We should finish our consultancy in 3–4 sentences. If I keep giving health education, I will have to close my clinic* (laughs)*. So I refer them to government, so that they don’t come back to me again”* [Minimally- Referring PP, Urban, General Practitioner].

Informal providers’ decision to refer patients to the RNTCP was dependent mostly on the infrastructure they possessed. PPs having small clinics referred their cases to the RNTCP because, of lack of knowledge about TB, fear of spread of infection in their clinic and availability of free treatment in the RNTCP. But the informal PPs owning nursing homes preferred to retain patients and they treated them for three to 4 months with private TB drugs.

We also observed a tendency among minimally-referring and non-referring PPs to put the blame on the patients or the poor functioning of government hospitals, as a reason for patient’s unwillingness to go to RNTCP as per their advice. A quote:

“*Government doctors do not treat patients well. The relationship between the patient and private practitioner is better compared to that of the government doctor and patient. That is the basic principle you should understand*” [Non-Referring PP, Rural, General Practitioner].

As a result of the above-described PP’s trade-offs, patient’s itineraries became complex. It was our consistent finding that once patients reached the RNTCP, treatment was started within a week in all cases.

#### Provision of kickbacks to PPs

When PPs were asked whether they receive any kickbacks from private laboratories for referring patients there, majority of PPs denied receiving kickbacks and some specialists were even offended by the question. However on further probing, few (*n* = 8) revealed that private laboratories gave kickback of around 5–10%, which they perceived to be meagre compared to the big labs located in big cities, where it could be as high as 30–40%. An excerpt:

“*Nothing… they are* [private labs] *just giving it* [kickbacks] *for the sake of it. It is not like Bangalore where they give 30% or 40%. Here* [Tumkur city] *they give 5 or 10%... it is nothing”* [Minimally-Referring PP, Urban, Specialist].

It appeared that the phenomenon of kickbacks was a norm in the private health sector and was available even to unqualified informal providers. An excerpt:

“*That is usual no madam...For X-ray, they* [private labs] *give 120 rupees* [1.8USD] *per patient referred. For sputum examination, they charge 50–60 rupee* [1USD] *per patient. So we hardly get anything there. Pharmacies… not really. They usually give us some gifts. You know how it works* (laughs)*”* [Non-Referring PP, Rural, Informal provider].

#### Patient’s search for relief

If we consider all participants, except four patients (patients D1, D3, D5 and R7), all other patient’s first encounter was with either private chemists or PPs. Since symptoms were not resolved, patients were forced to switch from one provider to another, concurrently and/or iteratively from public and private providers, leading to important diagnostic and treatment delays. An excerpt:

“*First they* [PPs] *told me to get X-ray, then they told me that there was no problem. I threw that report... I kept on visiting doctors and getting tested. I even got my blood and urine tested, but I was informed that there was no problem. I threw those reports too. I have almost 300 different bottles of syrup at home*” [Man, Rural, PP referred].

Patients expressed their wish to get more information about the disease from PPs. They were disappointed about provider’s attitude and inadequate communication during consultations. We found most of the PPs, especially the non-referring ones, did not regard the counselling and conveying provisional diagnosis of TB to patients as something that was paying off for them. They rather kept patients uninformed about the provisional diagnosis of TB to avoid offending them. PPs reported that, revealing the diagnosis of TB is a sensitive issue and patients could feel insulted, if the diagnosis of TB was revealed to them up-front and they might lose patients. Instead they preferred to refer them to any higher centres. An excerpt:

*“Many times even though I know this is a clear case of TB, I will not reveal it to patients. You see, all these are sensitive issues. So, why should I take the risk? So, I make statements in such a sensitive way that I don’t lose patients. So I persuade them go elsewhere”* [Minimally-Referring PP, Urban, General Practitioner].

## Discussion

We found patients’ awareness about TB was limited. Even though the symptoms were well experienced, patients did not relate them to TB. A first group of patients, self-medicated by purchasing medicines from private chemists for a long period, before seeking care. A second group sought care from government facilities and had simple itineraries. A third group, who sought care from PPs, switched concurrently and/or iteratively from public and private providers in search for relief of symptoms causing important diagnostic delays. Eventually all patients reached RNTCP and were started on treatment. PP’s cross-referral practices were influenced by patient’s paying capacity, familiarity with RNTCP, kickbacks from private labs and chemists, and in some cases, to get rid of TB patients. These trade-offs by PPs complicated patient’s itineraries to RNTCP. Patients were disappointed about providers’ attitude and inadequate communication during the consultations.

Currently RNTCP follows ‘passive case finding approach’. For this strategy to work effectively, patients need to have enough knowledge to understand that they need to seek care and where in the health system this is offered. Majority of TB patients in our study had little awareness about TB before getting diagnosed with it. A study in rural India that investigated the factors associated with patient delay, attributed it to the lack of patient awareness about TB [20], a finding that is confirmed by a nation-wide cross-sectional household survey in India [21]. These findings call for an innovative health education strategy in RNTCP to increase patient’s TB awareness, especially so with rural and less educated people. In the given scenario, community engagement becomes crucial to achieve the Government’s vision of ‘sweep out TB’ as envisioned in its national strategic plan (NSP) of TB elimination (2017–2025). Currently systematic efforts in this direction seem to be inadequate.

About half of the patients in our study self-medicated in the initial course of illness by purchasing drug over-the counter from retail private chemists. This resonates the findings from other studies which demonstrated the importance of retail private chemists as first-contact health care providers [22, 23] and the missed opportunities of utilising chemist’s services for early detection of TB cases [24]. Though RNTCP envisages a partnership with private chemists [25], institutionalising the processes of engaging them in RNTCP is yet to get established. Intervention research to improve the referrals from chemists to the RNTCP could contribute to the build-up of this knowledge.

Our study revealed low index of suspicion of TB among PPs. This corroborates the study findings that demonstrated sub-optimal TB management practices among PPs in India [26]. Detection of smear positive TB cases by sputum examination is a key element of DOT. Our study PPs, especially the minimally-referring and non-referring PPs ordered tests, which were not specific for TB, could have delayed the diagnosis of TB. Supporting this fact, a systematic review has shown that initially seeking care from a PP was clearly a significant risk factor for diagnostic delay [27]. Uplekar et al. in his study [28] showed that many PPs referred patients to government hospitals only late in the course of the patient’s illness. As demonstrated in our study, PPs are the one who decide on the type of the diagnostic tests and medications, thus wield considerable power. PP’s considerations for cross-referring cases to RNTCP were influenced by patient’s paying capacity, familiarity with RNTCP, kickbacks, and in some cases, to get rid of TB patients. These trade-offs by PPs had consequently complicated patient’s itineraries to RNTCP. It appears that PPs tend to see TB as source of income and not a public health responsibility. This could be corroborated with our finding which demonstrated the provision of kickbacks to PPs from private labs and chemists, a finding similar to a study from India [29]. RNTCP has made several policy changes mandating the notification of all TB patients from the private sector [30], establishing web-based TB surveillance system-NIKSHAY, ban on sero-diagnostics and amendments in H1 schedule to improve TB care services in private sector. But the response from the private sector to these policy changes has been poor [11]. Therefore, for effective implementation of these policies, a comprehensive, and system-oriented intervention has to be tested in the field settings, in collaboration with PPs.

We found in our study, in some instances the public sector providers were unable to diagnose TB. This is similar to the study findings from India that demonstrated the need for improvement in TB management practices in the public sector as well [31]. A recent study also found that, out of about 1.9 million TB patients who reached RNTCP in 2013, only about 1.6 million were successfully diagnosed with TB [32]. Further a recent study by Yellappa et al. has demonstrated how patients under RNTCP treatment had to cope up with stigmatisation, financial constraints and inconvenient DOT timings [16]. All these finding calls for urgent action to make RNTCP services more patients friendly within government facilities, to avoid TB patients getting deflected from RNTCP. India’s NSP proposes several bold strategies to prevent the loss of TB cases in the cascade of care. But these support systems should also expand to patients managed by private sector.

Best practices for TB diagnosis and treatment are enshrined in the Indian Standards of TB Care [33]. But, studies have shown that PPs in India do not often adhere to these standards [34]. A study from Indonesia has demonstrated that exposure of PPs to the National TB program improves referral of TB cases to National TB program [35]. In our study, all the actively-referring PPs were familiar and trusted the functioning of the RNTCP. Therefore, they counseled patients when they refused to go to the RNTCP. In contrast, non-referring and minimally-referring PPs had not undergone any formal RNTCP training and they doubted the efficiency RNTCP services. Hence, it is imperative that all providers irrespective of public or private should be sensitised about RNTCP services.

The majority of patients in our study initially sought care from primary level health care providers, who failed to diagnose TB. Hence, building the capacity of primary health care providers on better use of existing tests such as sputum examination, coupled with training them on TB symptoms is essential. WHO’s practical approach to lung health [36], which aims at improving the skills of primary health care workers, should be widely disseminated and implemented in India. Additionally, systematic referrals for sputum examination should be streamlined between providers and the RNTCP.

Alike other studies [37, 38], our study showed stigma surrounding TB still exists in the society and that revealing the provisional diagnosis of a stigmatised disease was perceived to be difficult by PPs [39]. This issue requires an attention, since poor communication and poor quality of information provided to patients could become an impediment to patient access to TB care [40]. Other study suggested that improved co-ordination between the PPs and the government health centres may substantially improve services for TB patients [41].

As an outcome of this study, an intervention has been developed to optimise the involvement of PPs and private chemists in the RNTCP. One set of interventions is targeted towards intervention private sector like training, improving communication between RNTCP staff and PPs, and other set of interventions is targeted towards general strengthening of RNTCP services [42].

The strength of this study is that it explains health-seeking trajectories from both patients’ and PPs’ perspective and helps to understand how TB patients navigate through the health facilities to seek care. Qualitative research method employed in this study allowed exploring patients’ perceptions and experiences about TB care, corroborated with PP’s TB management practices. We included a range of PPs providing TB care in different settings, which helped to identify their divergent perceptions and explore how they make decisions to refer patients to the RNTCP. This method of in-depth research to gather the narratives of TB patients and providers is useful for the programme managers to identify the weak links in the programme. However, the study findings must be interpreted with caution with regard to the specific study setting. Data was collected from such patients who accessed RNTCP. Therefore, the study does not provide any information about patients who did not reach RNTCP or died before. We have not considered patients who visited DMCs, but opted for treatment in the private sector. Documentation of patient itineraries described in the study is solely based on patient understanding and reconstruction of the events that occurred during the illness. Patients were identified from the RNTCP registers and they were initially contacted by RNTCP staff over phone to know their willingness to participate in the study. Hence, there are chances that some patients would have hesitated to share such information, which jeopardize their relationship with RNTCP staff. Patient narratives are based on reported historical events, which is vulnerable to recall bias.

## Conclusions

Government of India aims for universal good quality care for all TB patients in its national strategic plan, 2017–2025. In the backdrop of bold policy changes, our study findings may help RNTCP to develop initiatives to promote early detection of TB and develop supportive pathways to patient care. Our study highlights the critical role played by private sector, including informal providers and private pharmacists catering to TB patients and the dynamics around PP’s cross referral practices linked to the RNTCP. Patient’s and providers’ narratives from our study inform about the potential sources of delay in diagnosis and how better collaborations could be established with PPs within the realities of pluralistic health system of India. Our study has revealed the potential of private pharmacists in early TB case detection and the need for strengthening effective referral systems from private sectors to the RNTCP.

## Abbreviations

DOT: Directly observed treatment
NSP: National Strategic Plan
PP: Private practitioner
RNTCP: Revised National Tuberculosis Control programme
TB: Tuberculosis
